# Predicting cognitive impairment in outpatients with epilepsy using machine learning techniques

**DOI:** 10.1038/s41598-021-99506-3

**Published:** 2021-10-08

**Authors:** Feng Lin, Jiarui Han, Teng Xue, Jilan Lin, Shenggen Chen, Chaofeng Zhu, Han Lin, Xianyang Chen, Wanhui Lin, Huapin Huang

**Affiliations:** 1 Department of Neurology, Fujian Medical University Union Hospital, Fujian, People’s Republic of China; 2 BaoFeng Key Laboratory of Genetics and Metabolism, Beijing, People’s Republic of China; 3 Zhongguancun Biological and Medical Big Data Center, Beijing, People’s Republic of China

**Keywords:** Biotechnology, Neurology

## Abstract

Many studies report predictions for cognitive function but there are few predictions in epileptic patients; therefore, we established a workflow to efficiently predict outcomes of both the Mini-Mental State Examination (MMSE) and Montreal Cognitive Assessment (MoCA) in outpatients with epilepsy. Data from 441 outpatients with epilepsy were included; of these, 433 patients met the 12 clinical characteristic criteria and were divided into training (n = 304) and experimental (n = 129) groups. After descriptive statistics were analyzed, cross-validation was used to select the optimal model. The random forest (RF) algorithm was combined with the redundancy analysis (RDA) algorithm; then, optimal feature selection and resampling were carried out after removing linear redundancy information. The features that contributed more to multiple outcomes were selected. Finally, the external traceability of the model was evaluated using the follow-up data. The RF algorithm was the best prediction model for both MMSE and MoCA outcomes. Finally, seven markers were screened by overlapping the top ten important features for MMSE ranked by RF modeling, those ranked for MoCA ranked by RF modeling, and those for both assessments ranked by RDA. The optimal combination of features were namely, sex, age, age of onset, seizure frequency, brain MRI abnormalities, epileptiform discharge in EEG and usage of drugs. which was the most efficient in predicting outcomes of MMSE, MoCA, and both assessments.

## Introduction

Epilepsy is a disease resulting from abnormal synchronized neuronal discharge and is prone to influence cognitive function^1^. Approximately 30–40% of adult patients with epilepsy experience changes in cognition^2^. Many studies have suggested cognitive decline in memory, executive function, and attention in adult patients with epilepsy^3^. Meanwhile, some studies in the last decade have revealed that epilepsy and dementia have a common underlying etiological basis^4–7^. Moreover, it has been reported that some anticonvulsant drugs, such as valproate (VPA), may affect cognitive function in patients taking the drug^8,9^.

Most epilepsy patients are treated in outpatient clinics or emergency departments in China. Conventionally, Mini-Mental State Examination (MMSE) and Montreal Cognitive Assessment (MoCA) scales have gained popularity for cognitive screening. However, independent inspection and extra time are required to complete these scales. Early identification and rational management of epilepsy, including interventions, may reduce further impairment in cognitive function. Therefore, a fast and straightforward method to identify and predict cognitive function is urgently needed.

Machine learning is an artificial intelligence algorithm^10^ that helps clinicians make precise diagnoses and helps clinicians filter risk factors faster than ever^11–13^. For example, in a cross-sectional study, random forest (RF) regression analysis was used to investigate the relationship between daily total perceived data and MMSE scores^10^. However, the outcome of machine learning to predict cognitive function in epileptic patients is still unknown. In particular, MMSE and MoCA serve as screening tools, while the optimal features to predict either the MMSE or MoCA outcome are often not good for the other. A workflow to efficiently predict both MMSE and MoCA outcomes is important to determine the cognitive function in epilepsy patients at the first visit to the clinic.

In this study, we retrospectively reviewed clinical data of 433 consecutive outpatients with epilepsy. In the end, 304 cases were retained as the training dataset and 129 cases were designated as the validation dataset. Training dataset was used to better train stable, effective and reliable machine learning model; The validation dataset was to further verify and explore the generalization ability outside the model. First, the optimal model was selected by the machine learning method, and then the characteristic features with a high contribution rate were identified by the Youden index. Finally, redundancy analysis (RDA) combined with a machine learning algorithm achieved the optimal feature combination, and the optimal markers with RF exhibited the best prediction efficiency for the two outcomes of both the MMSE and MoCA. The results showed the highest classification accuracy of positive cases and the highest predictive power.

## Materials and methods

### Subjects and data preprocessing

The data of epileptic patients who consecutively visited the epileptic specialist clinic of the Fujian Medical University Union Hospital from January 1, 2015, to January 31, 2019, were retrospectively collected. This study was approved by the Union Hospital Ethics Committee (approval number: 2017KY085), and research involving human research participants must have been performed in accordance with the Declaration of Helsinki. All study subjects signed informed consent forms.

Inclusion criteria: Data were included for all patients equal to or older than 12 years. All patients matched the practical clinical definition of epilepsy established by the International League Against Epilepsy (ILAE) in 2014. All epileptic patients were examined by electroencephalogram (EEG), magnetic resonance imaging (MRI) and screening tools. Seizure types were categorized by the presence or absence of generalized tonic–clonic seizures tools clonic seizures. Positive family history was defined as a family history of epilepsy in the father, mother, sibling, or child of a participant. Brain trauma or surgery was determined according to past medical history. Epileptiform discharge was defined as a transient discharge obviously different from background activity in EEG recordings. The positive criterion for MRI was an abnormal focal signal increase or decrease. Positive electroencephalogram (EEG) results indicated that an epileptiform discharge was detected. Patients with primary epilepsy syndromes were excluded from this study.

Our study included patients who had undergone a neuropsychological outpatient assessment comprising MMSE and/or MoCA scales. Cognitive impairment was defined by MMSE scores ≤ 17 points (illiterate), ≤ 20 points (education level of primary school), or ≤ 24 points (education level of secondary school or above). MoCA scores ≥ 26 were considered to indicate normal cognition.

### Feature selection

We aimed to select common features that could be used to predict cognitive function. The characteristic features selected included the following demographic information and clinical features: sex (female/male), Seizure frequency, frequent—twice every six months; Occasional—semiannually; Rare—more than once a year, Seizure type—status epilepticus, negative ≤ 5 min; positive > 5 min; family history of epilepsy, febrile convulsion, and seizures, Seizure types were categorized by the presence or absence of generalized tonic–clonic seizures, status epilepticus (negative/positive), family history (negative/positive),positive family history was defined as a family history of epilepsy in the father, mother, sibling, or child of a participant., history of brain trauma or surgery (negative/positive).Brain trauma or surgery was determined according to past medical history., intracerebral diseases (negative/positive), lesions in brain MRI (negative/positive),The positive criterion for MRI was an abnormal focal signal increase or decrease, epileptiform electroencephalogram (EEG) discharges (negative/positive),positive electroencephalogram (EEG) results indicated that epileptiform discharge was detected., and usage of valproate sodium (yes/no). The measurement data included the patients’ current age and age of onset (years).The original dataset was randomly divided into a training dataset (70%) and a validation dataset (30%). The training data set included 304 cases, and the test data set included 129 cases. The classified features in the included data were analyzed. The measured data are represented by the mean ± SD.

### Data preprocessing

**Figure 1:**
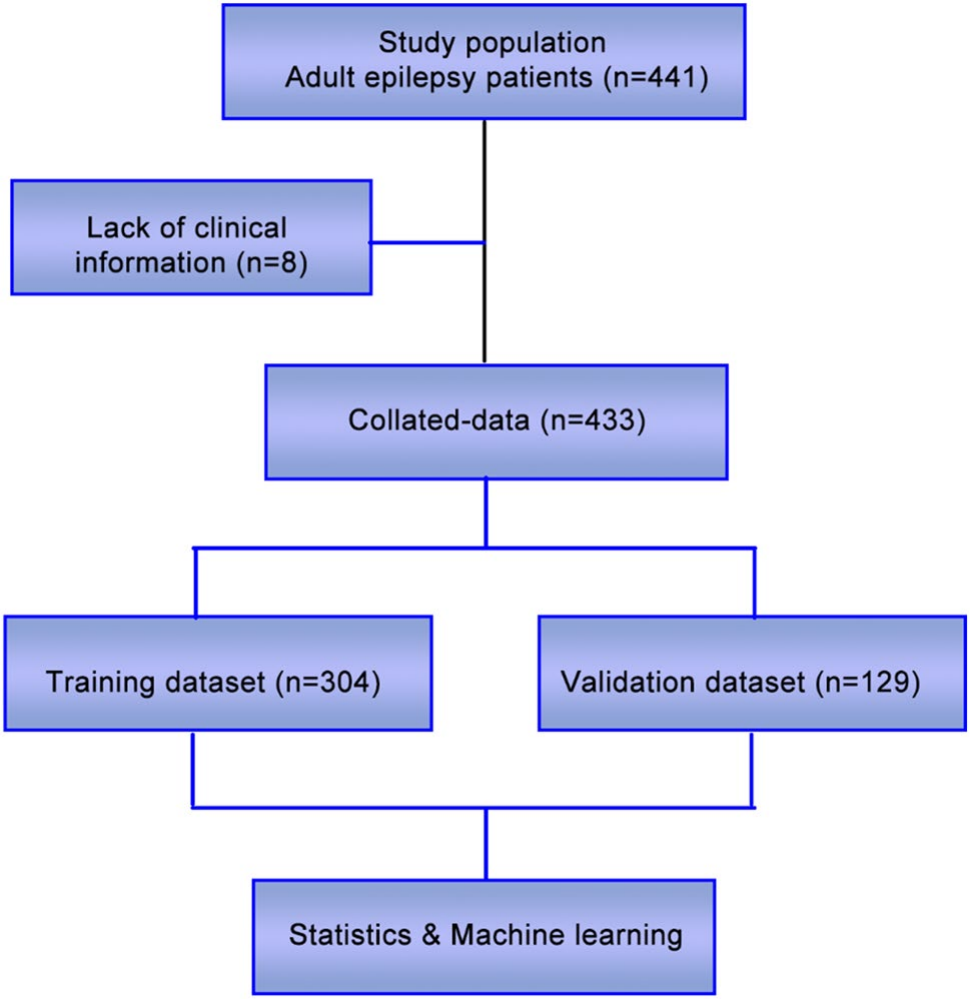
Clinical data screening process.

All patient data collected included both MMSE and MoCA scores. Data lacking MMSE or MoCA scores were screened out. Data with incomplete information were removed (Fig. 1). A set of observation features was selected to predict the cognitive function of these patients by a machine learning method, and a model was established to estimate the rank of features that predict cognitive function.

### Machine learning-based classification

All statistical analyses were conducted using R (US version 3.6.3) and R studio (US version 1.1.456). If there were missing values, the median was used to interpolate classified features, and the mean was used to interpolate continuous features.

Before binary classification, tenfold cross-validation was carried out to select the optimal prediction model for MMSE. We used four machine learning algorithms: logistic regression (LR), DT, RF and SVM. Each model’s accuracy, positive predictive value, and specificity are described. The area under the curve (AUC) and the optimal threshold were determined by the receiver operating characteristic (ROC) curve.After the optimal model was selected, the first ten features with high contributions to the model were selected. At the same time, the original data were analyzed by RDA, the first ten characteristic features were selected, the features selected by the two methods were intersected, and the Monte Carlo method was used to verify the internal tenfold cross-validation of cognitive impairment in patients with epilepsy. Then, the test set was used to verify outcomes. Before analysis, data from derived sets were randomly divided into separate training and validation datasets, comprised of 70% and 30% of the cases, respectively. All meaningful tests are bilateral. *P* < 0.05 was statistically significant.

### Model evaluation

To evaluate the performance of the training model, we used the following representative indicators: sensitivity, specificity, accuracy, precision, recall, and AUC. The greater the value of sensitivity, the more the “sick individuals were considered to be sick.” At the same time, a more significant value of specificity indicated that “healthy individuals were considered to be healthy”. Accuracy refers to the proportion of the correctly predicted samples to the total predicted samples. Precision refers to the ratio of correctly predicted positive samples to all predicted positive samples. Recall refers to the ratio of correctly predicted positive samples to the total number of true positive samples. The AUC of a classifier is equivalent to the probability that the classifier will rank a randomly chosen positive instance higher than a randomly chosen negative instance^14^. From a clinician’s perspective, high accuracy means that our predictions are rarely overreported and indicates that patients are more likely to have cognitive impairment. At the same time, a high recall rate indicates that many positive examples in the sample are predicted correctly.

### Ethics approval

This study was approved by the Union Hospital Ethics Committee (Approval Number: 2017KY085), and all study subjects signed informed consent forms.

## Results

### Patient demographics

In order to make the influence of missing data reach a relatively unbiased and controllable state after the imputation method was applied, eight cases with over 30% missing clinically relevant information from a total number of 441 were excluded^15^. A total of 433 patients with complete information were analyzed and divided into a training group (n = 304) and a test group (n = 129). Data were included for all patients equal to or older than 12 years. The results of Wilcoxon rank sum test of age showed that the grouping of MMSE was different (training group: W = 5378, *p* = 1.295E-05; test group: W = 927.5, *p*-value = 0.003), there is no difference for MOCA (training group: W = 9284.5, *p*-value = 0.290; test group: W = 1595, *p*-value = 0.244). According to this result, we did not target age groups, and multivariate statistics can analyze the statistical laws of multiple objects and indicators when they are correlated with each other.

The database consisted of 12 features, including two different categories, in which two features provided demographic information and the other 10 features provided clinical symptom features used to monitor clinical outcomes. Descriptive statistical analysis was performed on 12 clinical features. Table 1 showed the details of the selected features.

**Table 1:** Features of epileptic patients.

Features	N (%) or mean ± SD
**Sex**	
Female	194 (44.80%)
Male	239 (55.20%)
Age	26.00 ± 4.24
Age of onset	24.50 ± 2.12
**Seizure frequency**	
Frequent	75 (17.32%)
Occasional	180 (41.57%)
Rare	178 (41.11%)
**Seizure type**	
Without generalized tonic–clonic seizures	142 (32.79%)
With generalized tonic–clonic seizures	291 (67.21%)
**Status epilepticus**	
Negative	417 (96.30%)
**Positive**	16 (3.70%)
**Family history**	
Negative	415 (95.84%)
Positive	18 (4.16%)
**History of brain trauma or surgery**	
Negative	365 (84.29%)
Positive	68 (15.71%)
**Other brain diseases**	
Negative	390 (90.06%)
Positive	43 (9.94%)
**Brain MRI abnormalities**	
Negative	333 (76.90%)
Positive	100 (23.10%)
**Epileptiform discharge in EEG**	
Negative	74 (17.09%)
Positive	359 (82.91%)
**Usage of drugs**	
Valproate sodium (1)	136 (31.40%)
No drugs (2)	23 (5.31%)
Others (3)	274 (63.29%)

### Prediction modeling for MMSE scores of patients with diagnosed epilepsy

In total, there were 304 patients in the training dataset. We identified patients with cognitive impairment using the MMSE scale according to educational level. Seventy patients with epilepsy were found to have cognitive impairment. logistic regression (LR), DT, RF and SVM model’s accuracy, positive predictive value, and specificity are described in Table 2. The AUC values of these four models (LR, DT, RF, and SVM) were 0.67, 0.63, 0.72 and 0.70, respectively. Meanwhile, the mean AUC after internal cross-validation within RF modeling, which was 0.72, was significantly higher than that of the other models. The RF was selected as the optimal modeling approach.

**Table 2:** Results of cross-validation for different machine learning algorithms using MMSE data.

Model	Sensitivity	Specificity	Accuracy	Precision	Recall	AUC
LR	0.68	0.75	0.73	0.48	0.68	0.67
DT	0.41	0.89	0.78	0.70	0.41	0.63
RF	0.69	0.80	0.78	0.63	0.69	0.72
SVM	0.60	0.90	0.83	0.73	0.60	0.70

Furthermore, we obtained the ranking of the importance of each feature in the RF model. The top ten features in the ranking of feature importance were age, age of onset, sex, usage of drugs, history of brain trauma or surgery, seizure type, seizure frequency, epileptiform discharge in EEG, brain MRI abnormalities, and other brain diseases. (Table 3). Mean decrease in accuracy, the higher the value, the greater the importance of the variable.

**Table 3:** Ranking of feature importance in the RF model (MMSE).

	CK	DIS	Mean decrease in accuracy
Age	12.12	12.67	13.78
Age of onset	10.76	10.94	12.92
Sex	4.85	4.70	6.18
Usage of drugs	3.13	3.50	4.51
History of brain trauma or surgery	3.12	1.62	3.39
Seizure type	2.91	0.70	2.75
Seizure frequency	2.26	− 1.35	1.17
Epileptiform discharge in EEG	2.34	− 1.40	1.12
Brain MRI abnormalities	0.49	0.70	0.82
Other brain diseases	0.41	0.61	0.64
Family history	− 0.90	2.39	0.53
Status epilepticus	− 0.67	0.41	− 0.25

*CK* control check; *DIS* disease—people with epilepsy.

### Prediction modeling for MoCA scores of patients with diagnosed epilepsy

We used the same strategy described above to build four common machine learning models. Our results showed that the AUC values of these four models (LR, DT, SVM, and RF) were 0.62, 0.61, 0.60, and 0.71, respectively (Table 4). Therefore, in terms of generalization of the different machine learning algorithms, the RF model achieved an AUC of 0.71; thus, it performed better than others using MoCA data.

**Table 4:** Results of cross-validation for different machine learning algorithms using MoCA data.

Model	Sensitivity	Specificity	Accuracy	Precision	Recall	AUC
LR	0.57	0.76	0.64	0.84	0.57	0.62
DT	0.73	0.53	0.67	0.78	0.73	0.61
SVM	0.66	0.76	0.69	0.87	0.66	0.60
RF	0.51	0.81	0.60	0.88	0.51	0.71

We determined the variable importance in the RF model. The top ten features in the ranking of feature importance were status epilepticus, history of brain trauma or surgery, seizure frequency, sex, usage of drugs, age, epileptiform discharge in EEG, family history, brain MRI abnormalities and age of onset (Table 5).

**Table 5:** Ranking of feature importance in the RF model (MoCA).

	CK	DIS	Mean decrease in accuracy
Status epilepticus	− 3.27	− 2.31	3.66
History of brain trauma or surgery	0.90	3.32	3.09
Seizure frequency	1.27	1.97	2.46
Sex	− 2.46	4.65	2.39
Usage of drugs	0.26	2.96	2.30
Age	2.35	0.6	1.78
Epileptiform discharge in EEG	− 1.95	− 0.87	1.68
Family history	1.01	1.52	1.62
Brain MRI abnormalities	1.28	0.13	0.90
Age of onset	− 1.07	1.49	0.71
Other brain diseases	1.72	− 0.56	0.56
Seizure type	− 2.86	1.69	0.34

### RDA contributes to variable constraint

**Figure 2:**
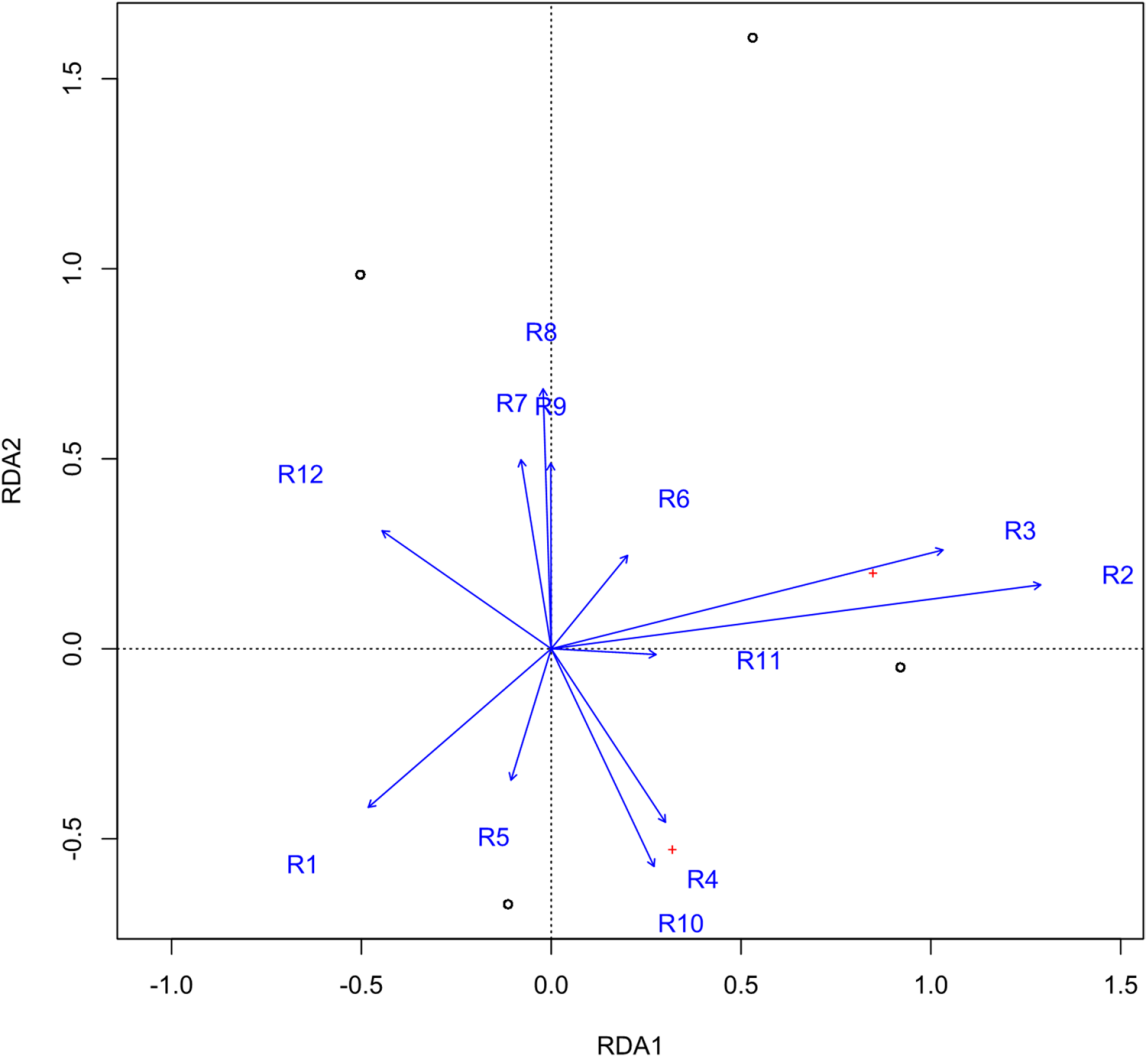
RDA analysis plot. The length of the arrow shows the strength of the correlation between the variable and the result variable. The longer the arrow length, the stronger the correlation. The vertical distance reflects the correlation between them. The smaller the distance, the stronger the correlation.

Unlike other single outcome features, RDA can explain the comprehensive relationship between dual outcomes and exposure features. Then, the degree that the critical features explain the double outcome can be determined, and the redundant information can be removed. Therefore, the RDA model was used in this study as a method to study the correlation between the outcome variable matrix and the exposure variable matrix. Eigenvalues of RDA1 and RDA2 were 0.719 and 0.281, respectively. The accumulated constrained eigenvalues showed the contribution of features (Table 6, Fig. 2).

**Table 6:** The contribution of features in the RDA model.

	RDA1	RDA2
Age	0.86	0.11
Age of onset	0.69	0.17
Sex	− 0.32	− 0.27
Usage of drugs	− 0.29	0.20
Seizure frequency	0.20	− 0.30
Epileptiform discharge in EEG	0.18	− 0.01
Brain MRI abnormalities	0.18	− 0.38
Status epilepticus	0.13	0.16
Seizure type	− 0.07	− 0.23
Family history	− 0.05	0.33
History of brain trauma or surgery	− 0.01	0.45
Other brain diseases	− 0.00	0.32

The features of the top ten contribution rates for MMSE and MoCA outcomes were age, age of onset, sex, usage of drugs, seizure frequency, epileptiform discharge in EEG, brain MRI abnormalities, status epilepticus, seizure type and family history ranked by RDA1 values.

### Selection of the optimal combination of features

**Figure 3:**
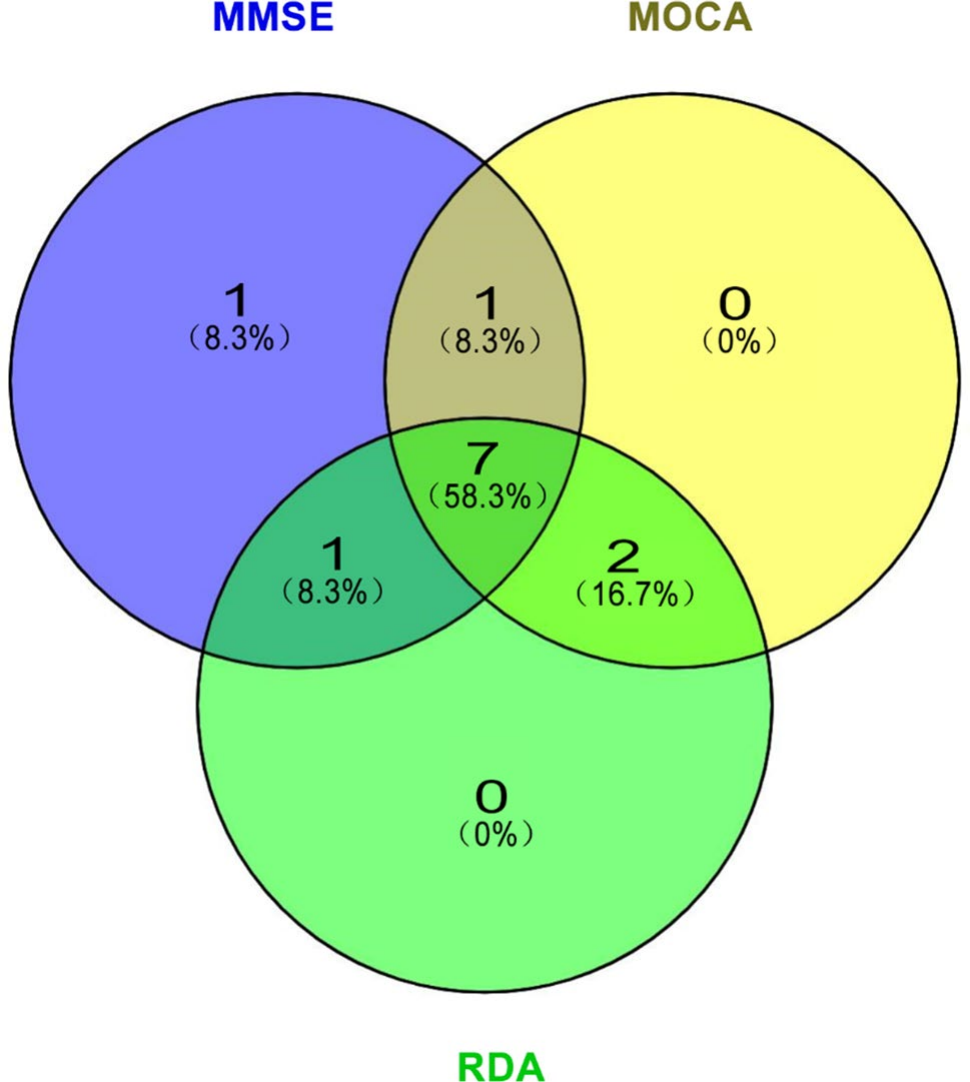
In the Venn diagram, each circle represents the difference variable in a model, the number of overlaps in the circle represents the number of common variables in the two models, and the overlap area represents the number of unique variables in each model (purple: MMSE; Yellow: MOCA; Green: RDA).

In the RF modeling of MMSE or MoCA data, we obtained the top ten characteristic variables with contribution rates. Additionally, we selected the top ten features according to the contribution rate with bivariate outcomes from the RDA model. The optimal candidate features were filtered by Venn analysis, and there were 7 overlapping features, namely, sex, age, age of onset, seizure frequency, brain MRI abnormalities, epileptiform discharge in EEG and usage of drugs (Fig. 3).

### Validation for the optimal combination of features

To determine the optimal combination of features, we chose the optimal combination of features through the optimal model for internal validation of binary classification, the top ten features of RDA modeling, the top ten features of MoCA outcomes in RF modeling, and the top ten features of MMSE outcomes in RF modeling for external validation.

### Validation for MMSE outcomes

**Figure 4:**
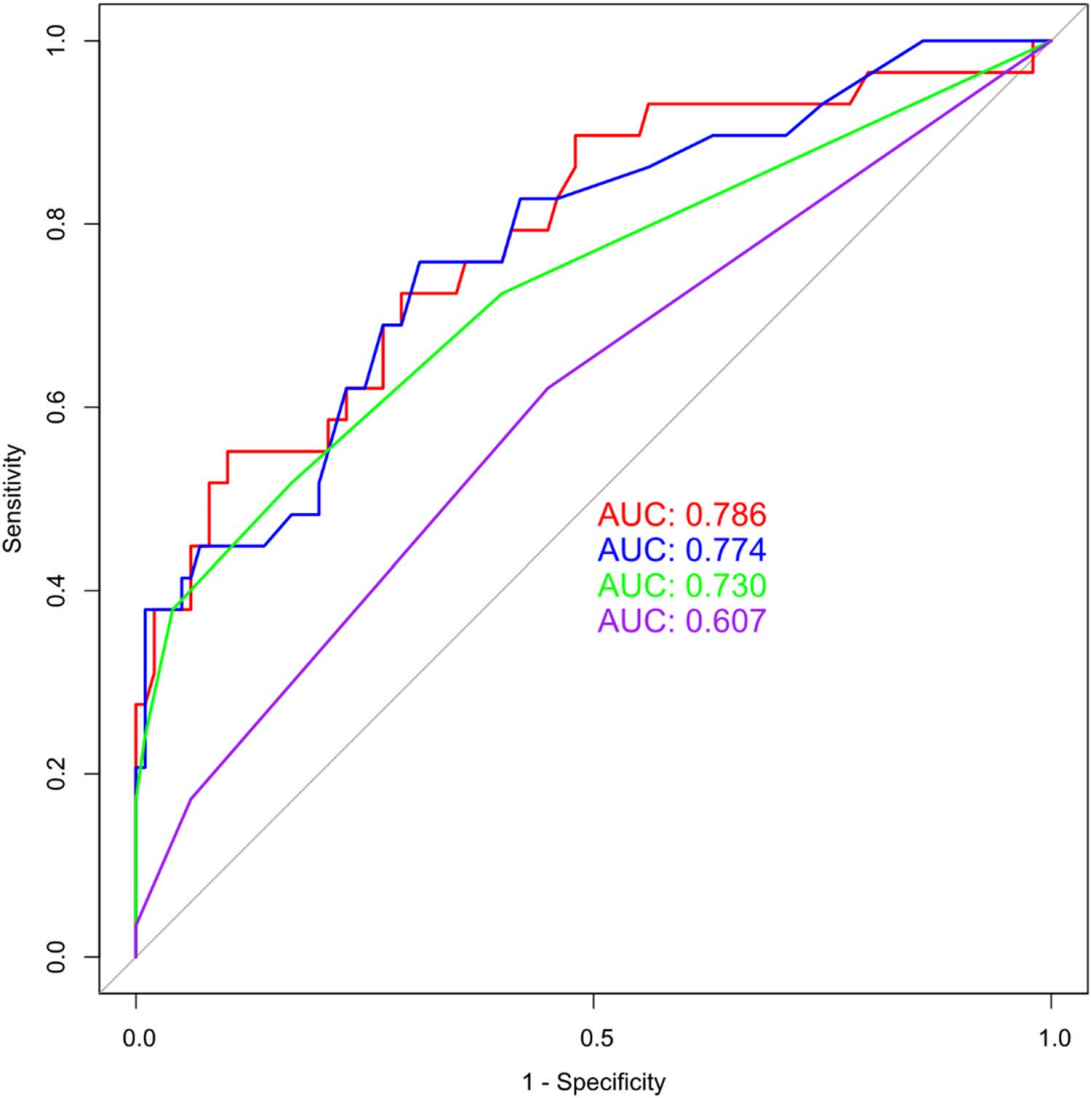
ROC curve of MMSE's prediction model. (red: the optimal combination of variables; blue: the top ten features of RDA; green: the top ten features of MMSE RF analysis; purple: the top ten features of MoCA RF analysis).

Verification results of various variable combinations for RF models showed that the ROC value of the optimal combination of features, which was 0.786, was the highest (Fig. 4). After analyzing all the combinations of features details, the optimal combination of features revealed that highest candidate variable combinations had specificity, accuracy, and precision values of 0.90, 0.82, and 0.61, respectively (Table 7).

**Table 7:** Validation dataset validated all the combinations of features (MMSE).

Model	Susceptibility	Specificity	Accuracy	Precision	Recall
The optimal combination of features	0.55	0.90	0.82	0.61	0.55
The top ten features of RDA	0.76	0.69	0.70	0.4	0.76
The top ten features of MMSE RF analysis	0.52	0.83	0.76	0.47	0.52
The top ten features of MoCA RF analysis	0.62	0.55	0.56	0.28	0.62

*MMSE* Mini-Mental State Examination; *MoCA* Montreal Cognitive Assessment.

### Validation for MoCA outcomes

**Figure 5:**
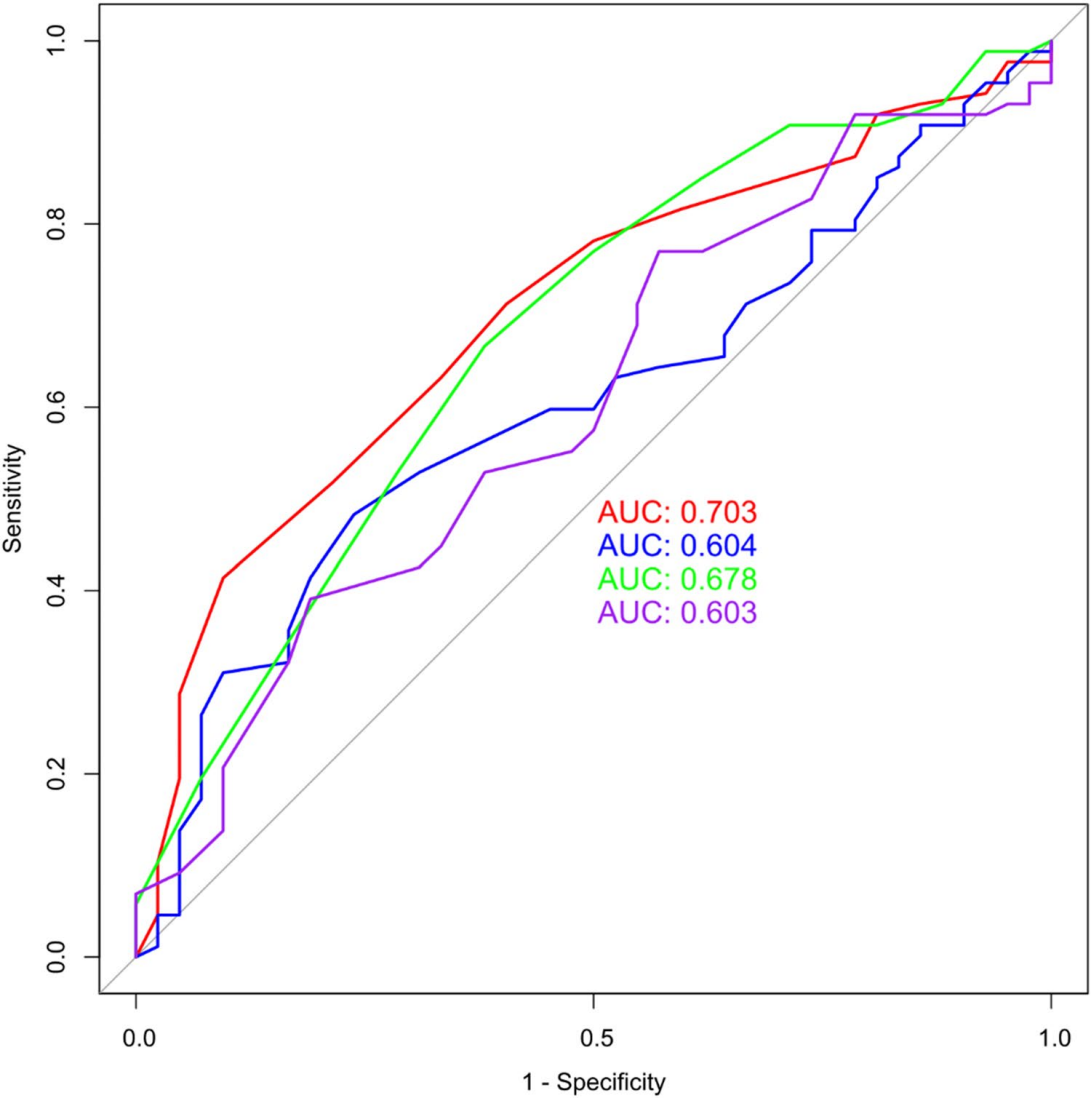
ROC curve of MOCA's prediction model. (red: the optimal combination of variables; blue: the top ten features of RDA; green: the top ten features of MMSE RF analysis; purple: the top ten features of MoCA RF analysis).

Verification results of various variable combination models showed that the ROC value of the optimal combination of features, which was 0.702, was the highest (Fig. 5). All the combinations of features in detail and the specificity and precision of the optimal combination of features, which were 0.90 and 0.90, respectively, were also the highest (Table 8).

**Table 8:** Validation dataset validated all the combinations of features (MoCA).

Model	Susceptibility	Specificity	Accuracy	Precision	Recall
The optimal combination of features	0.41	0.90	0.57	0.90	0.41
The top ten features of RDA	0.48	0.76	0.57	0.81	0.48
The top ten features of MMSE RF analysis	0.67	0.62	0.65	0.78	0.66
The top ten features of MoCA RF analysis	0.39	0.81	0.53	0.81	0.39

### Evaluation for dual MMSE and MoCA outcomes

Different candidate variables had different clinical values. The optimal combination of features was used not only to predict MMSE and MoCA scores but also to predict the results of double outcome variables. We plotted the mixed matrix of four combinations predicting two MMSE and MoCA outcomes and calculated the accuracy (Table 9). The column names in the table are expressed as MMSE outcomes (0 indicates normal and 1 indicates cognitive function) and MoCA outcomes (0 indicates normal and 1 indicates cognitive function).

**Table 9:** Different candidate variables predict the correct probability at the same time.

Model	(0, 0) (%)	(0, 1) (%)	(1, 0) (%)	(1, 1) (%)
The optimal combination of features	38.10	70.00	21.43	50.00
The top ten features of RDA	38.47	72.97	13.16	46.67
The top ten features of MMSE RF analysis	16.67	28.57	0	4.88
The top ten features of MoCA RF analysis	10.00	39.53	4.55	9.09

The results showed that the best predictor of noncognitive function (MMSE = 0, MoCA = 0) was the top ten features of RDA, followed by the candidate variable combination; prediction accuracy was 38.47% and 38.1%, respectively. In particular, the best predictor for cognitive function (MMSE = 1, MoCA = 1) was the optimal combination of features, and the accuracy was 50.00% (Table 9). All the results above indicate that candidate features were the optimal combination of features not only for the prediction of MMSE or MoCA outcomes individually but also for both.

## Discussion

Epilepsy is a common neurological disease and is associated with impairments in cognitive function. There are several factors related to cognitive comorbidities, such as disease duration, seizure types, usage of VPA, and others. However, cognitive function prediction lacks a valid and convenient tool that could be used in clinical practice until now. We used retrospective clinical data to construct a promising prediction model of MMSE and MoCA outcomes using machine learning.

In our study, 12 features were used for statistical analysis and modeling. We found that the RF algorithm is the most effective model to predict cognition outcomes in this kind of patient. Significant features, such as sex (R1), age (R2), age of onset (R3), seizure frequency (R4), brain MR focus (R10), EEG (R11), and drugs (R12), were screened out. Interestingly, correlation analysis showed no significance for these factors. This may be due to the heterogeneity of the study population in the real world and the small sample size.

The order of significant features is different between the MMSE and MoCA scales. For example, MRI abnormalities and age at onset (AAO) are important in MoCA outcomes, and sex and seizure types are important in MMSE outcomes, while age is important for both scales. These results are partly consistent with previous studies^16–18^. This could be due to two scales focusing on the different aspects of function. The MMSE focuses on memory, while the MoCA focuses on executive function^19^. It is possible that different factors may contribute to various aspects of cognitive function decline^20^. Jan Bressler et al. analyzed the relationship between age acceleration, and cognitive function using three neuropsychological tests that represent different cognitive domains that decline with age to different degrees^21^, and cognitive function declines with age^22–24^. At the same time, the frequency of seizures is also strongly linked to cognitive function. Previous studies have shown that the rapid decline in cognitive function in patients with seizures may be associated with seizures^25,26^. Frequent seizures can also interfere with normal neuronal physiology and brain development, disrupt various neurocognitive processes (such as plastic memory encoding and language processing), and cause developmental delay, regression, or interruption. For instance, Landau-Kleffner syndrome is characterized by language degradation in normally developing children and is associated with changes in electroencephalography, mainly during sleep^27^. Cognitive function in patients with epileptic encephalopathy was improved after normalization of EEG^28^.

Until now, most of the exploration in epilepsy with cognitive impairment has used traditional advanced statistical methods, such as LR^29^. These methods have an overt restriction on the sample size of the research population. These methods are not easy in clinical application because the results vary in age and clinical phenotype. Moreover, traditional methods cannot predict dual MMSE and MoCA outcomes. Our study found an approach to efficiently predict the dual outcome of MMSE and MoCA assessments.

A method based on machine learning has been applied to in the field of epilepsy research to a certain extent^30–32^. In the present study, we established a workflow to efficiently predict dual outcomes of the MMSE and MoCA in outpatients with epilepsy. Obviously, compared with the traditional method of directly eliminating excess factors, the introduction of RDA can undoubtedly improve the screening accuracy of the model for potential patients, and the correct rate can be improved to 50%. The main reasons may be due to the stable internal verification premise, the clear choice of modeling algorithm, the effective control of redundant information, and the balance adjustment of model parameters. First, we selected a series of necessary candidate features to distinguish the different cognitive levels of patients with baseline epilepsy. These features include age, age of onset and seizure frequency. Whether VPA was used previously is also an important factor affecting cognitive function. Second, we carried out robust internal verification to compare the stability of traditional linear algorithms and machine learning nonlinear algorithms. Then, the characteristics that contribute more to multiple outcomes were screened out through the variable importance of the unsupervised learning model and the variable importance of the restricted model. In particular, the RF algorithm was combined with the redundancy analysis algorithm. After the linear redundant information was removed, optimal feature selection and resampling were carried out. Finally, the follow-up data were used to backtrack the model, further confirming the external rigor and predictive efficiency of the workflow. Therefore, we output a more accurate classifier based on a supervised learning algorithm and linear constraint algorithm for early screening of cognitive impairment in patients with epilepsy.

This study was the first to use machine learning to predict cognitive functions in specific epilepsy categories so far. More importantly, we have proposed a method for predicting dual outcomes that can be used with other prediction models in other diseases. However, this study included only a small sample of adolescents patients and did not include patients under 12 years of age, which is a result of the nature of our neurology department, which emphasizes adult patients. The lack of complete outpatient data leads to a significant decrease in the number of patients enrolled in this study. From a clinical point of view, it would be more relevant to know the contribution of common different antiepileptic medications to the model. Data processing A more effective prediction model could be created by expanding the sample size in the future.

Our study addressed the statistics of features in the real world, which has high heterogeneity. This prediction using a variable combination with machine learning could be used in the research of heterogeneous epileptic patients. This approach may help clinical experts conveniently identify and avoid impairment of epilepsy patients’ cognitive function by determining patients’ cognitive functions early.

## Conclusion

Our research clarified that the cognitive function of patients could be predicted by common clinical information via machine learning. Moreover, we found that our optimal variable combination with RF modeling is the best way to predict the cognitive function of outpatients with epilepsy. This can help clinicians assess outpatients’ cognitive function and prevent further damage at the first visit.

## Data Availability

The corresponding author had full access to all the data in the study and take responsibility for the integrity of the data and the accuracy of the data analysis.
